# Development of an Innovative Biosensor Based on Graphene/PEDOT/Tyrosinase for the Detection of Phenolic Compounds in River Waters

**DOI:** 10.3390/ijms25084419

**Published:** 2024-04-17

**Authors:** Alexandra Virginia Bounegru, Catalina Iticescu, Lucian P. Georgescu, Constantin Apetrei

**Affiliations:** Department of Chemistry, Physics and Environment, Faculty of Sciences and Environment, “Dunărea de Jos” University of Galati, 47 Domneasca Street, 800008 Galați, Romania; alexandra.meresescu@ugal.ro (A.V.B.); catalina.iticescu@ugal.ro (C.I.);

**Keywords:** catechol, biosensor, sensitivity, selectivity, square wave voltammetry

## Abstract

Phenolic compounds, originating from industrial, agricultural, and urban sources, can leach into flowing waters, adversely affecting aquatic life, biodiversity, and compromising the quality of drinking water, posing potential health hazards to humans. Thus, monitoring and mitigating the presence of phenolic compounds in flowing waters are essential for preserving ecosystem integrity and safeguarding public health. This study explores the development and performance of an innovative sensor based on screen-printed electrode (SPE) modified with graphene (GPH), poly(3,4-ethylenedioxythiophene) (PEDOT), and tyrosinase (Ty), designed for water analysis, focusing on the manufacturing process and the obtained electroanalytical results. The proposed biosensor (SPE/GPH/PEDOT/Ty) was designed to achieve a high level of precision and sensitivity, as well as to allow efficient analytical recoveries. Special attention was given to the manufacturing process and optimization of the modifying elements’ composition. This study highlights the potential of the biosensor as an efficient and reliable solution for water analysis. Modification with graphene, the synthesis and electropolymerization deposition of the PEDOT polymer, and tyrosinase immobilization contributed to obtaining a high-performance and robust biosensor, presenting promising perspectives in monitoring the quality of the aquatic environment. Regarding the electroanalytical experimental results, the detection limits (LODs) obtained with this biosensor are extremely low for all phenolic compounds (8.63 × 10^−10^ M for catechol, 7.72 × 10^−10^ M for 3-methoxycatechol, and 9.56 × 10^−10^ M for 4-methylcatechol), emphasizing its ability to accurately measure even subtle variations in the trace compound parameters. The enhanced sensitivity of the biosensor facilitates detection and quantification in river water samples. Analytical recovery is also an essential aspect, and the biosensor presents consistent and reproducible results. This feature significantly improves the reliability and usefulness of the biosensor in practical applications, making it suitable for monitoring industrial or river water.

## 1. Introduction

Water represents one of the most precious natural resources and is essential for supporting life on Earth. However, increasing industrialization, urbanization, and agricultural activities have led to water contamination with various toxic compounds and pollutants, including phenolic compounds. Phenolic compounds are organic compounds containing a phenolic functional group and can be found in water as a result of industrial processes, agricultural waste, or as degradation products of natural compounds [1]. These phenolic compounds hold significant importance in the environment due to their toxicity to ecosystems and human health [2]. Due to their environmental and health risks, phenolic compounds are regulated by organizations like the WHO and EPA, with thresholds ranging from 1 μg L^−1^ in drinking water to 0.03–4000 μg·L^−1^ in surface water [3]. However, these regulations often overlook certain phenolic compounds, highlighting the need for improved standards through environmental and health risk studies.

Detection and monitoring of phenolic compounds in surface and ground water thus become a priority in environmental protection efforts and ensuring the quality of drinking water. For instance, catechol (CAT), 3-methoxycatechol (3-methoxyCAT), and 4-methylcatechol (4-methylCAT) can contaminate both wastewater and surface water, resulting from industrial and agricultural waste discharges from manufacturing processes and the use of chemicals in practice. In this context, the development of sensitive, selective, and rapid methods for the analysis of phenolic compounds becomes crucial [4].

Classical methods of chemical analysis, such as liquid chromatography and mass spectrometry [5,6], are employed for the detection of phenolic compounds, but these often involve high costs, complex equipment, and prolonged analysis times. This renders these methods unsuitable for routine and on-site analyses.

In this context, electrochemical methods have gained increasing attention in recent years due to their advantages in detecting phenolic compounds [7,8]. These methods provide a rapid, sensitive, and convenient alternative for the analysis of phenolic compounds, using sensors modified with conductive polymers.

Sensors modified with conductive polymers represent an emerging technology that utilizes polymers with specific electrochemical properties to enhance sensor performance [9].

Graphene and conducting polymers, such as PEDOT, polypyrrole, and polyaniline, are examples of materials used in modifying sensors with remarkable results. These modified sensors exhibit a large surface area, high conductivity, and chemical stability, making them suitable for the detection of phenolic compounds in water [10,11,12,13].

Additionally, enzymatic biosensors represent another promising approach for the detection of phenolic compounds [14,15]. These biosensors use specific enzymes, such as tyrosinase, to catalyze the oxidation or reduction reactions of phenolic compounds. Due to the high specificity and sensitivity of enzymes, enzymatic biosensors can accurately and selectively detect phenolic compounds at very low concentrations [16,17,18].

Our previous experience with tyrosinase-based biosensors has demonstrated efficient results in detecting phenolic compounds. These enzymatic biosensors are capable of catalyzing the oxidation reactions of phenolic compounds, transforming them into intermediate or final products in the presence of tyrosinase. The electrochemical reactions resulting from these oxidation processes can be detected and measured using electrochemical methods such as cyclic voltammetry (CV), differential pulse voltammetry (DPV), and square wave voltammetry (SWV) [19,20,21].

In this work, we aim to develop and characterize an enzymatic biosensor based on a modified screen-printed electrode with graphene and PEDOT for the detection of low concentrations of phenolic compounds (CAT, 4-methylCAT, and 3-methoxyCAT in water samples. We will employ electrochemical methods such as cyclic voltammetry and electrochemical impedance spectroscopy to evaluate the performance of the newly developed biosensor. Additionally, we will compare the results of the sensitivity and precision of detection using the standard addition method and assess the stability and reproducibility of the biosensor. The novelty of this study lies in the implementation of rapid, simplified modification steps with optimized parameters, minimizing reagent consumption. This optimization of the manufacturing process has led to the development of an efficient enzymatic biosensor for the selective detection of phenolic compounds with chemical structures similar to catechol, 3-methoxycatechol, and 4-methylcatechol.

The objective of this work is to establish an efficient and sensitive method for detecting phenolic compounds in river waters, with the potential to enhance the monitoring and protection of the aquatic environment and public health. The development of a new enzymatic biosensor based on tyrosinase, graphene, and PEDOT may contribute significantly to the existing methods for detecting phenolic compounds in river waters.

The originality of this work lies in the innovative approach to developing a biosensor with remarkable selectivity for the detection and quantification of three phenolic compounds—CAT, 3-methoxyCAT, and 4-methylCAT—which are structurally very close. This structural similarity presents a significant challenge in the precise selection and detection of these compounds due to their chemical likeness. Adapting and optimizing the proposed biosensor (SPE/GPH/PEDOT/Ty) to achieve high selectivity in detecting these phenolic compounds with similar chemical structures represent a significant advancement in water analysis. This approach not only provides a reliable and efficient method for monitoring phenolic compounds in flowing waters and other water sources but also has significant implications for environmental protection and public health.

## 2. Results and Discussion

### 2.1. Surface Morphological Characterizations of SPE/GPH/PEDOT/Ty

**Figure 1:**
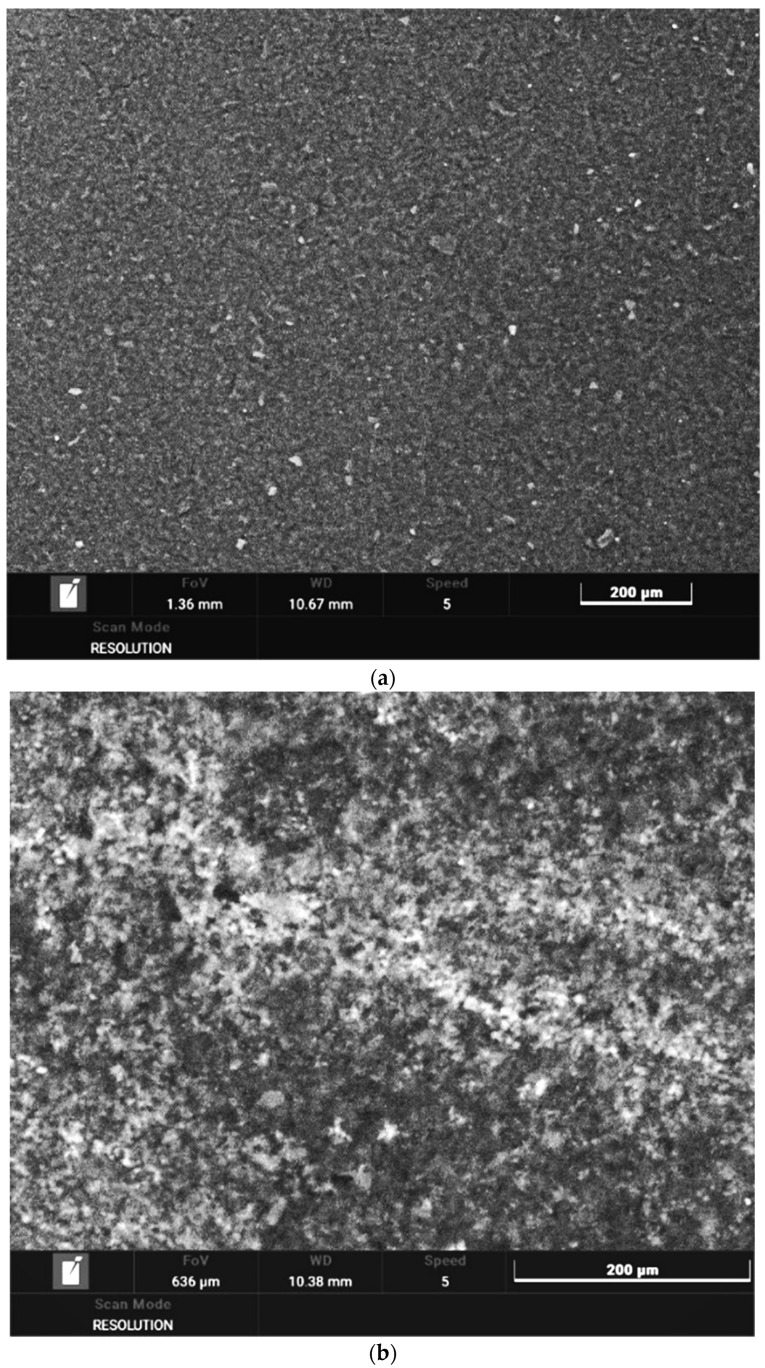

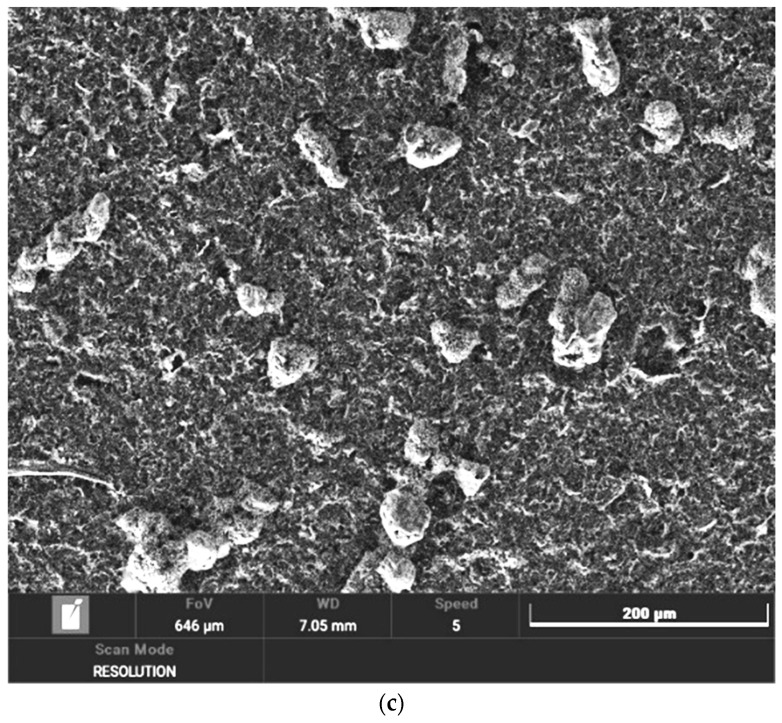
SEM pictures of (**a**) SPE/GPH, (**b**) SPE/GPH/PEDOT, and (**c**) SPE/GPH/PEDOT/Ty.

The detailed analysis of surface morphology through scanning electron microscopy (SEM) is an important and essential step in the development process of a high-performance biosensor. Therefore, an SEM analysis was conducted to evaluate the morphology of the SPE/GPH, SPE/GPH/PEDOT, and SPE/GPH/PEDOT/Ty substrates. In Figure 1a, the successfully deposited graphene layer on the SPE substrate surface is observed. This graphene layer appears as a smooth surface, indicating an efficient and uniform application of the material on the substrate. A smooth and uniform surface provides an ideal substrate for the uniform deposition of the PEDOT film, which can improve its adhesion and stability. This behavior has been reported in other studies as well [22,23]. The overall appearance is without visible defects or significant roughness, suggesting good deposition quality and a regular texture.

In Figure 1b, visualized through SEM, the PEDOT film overlaid on the graphene layer (SPE/GPH/PEDOT) is noticeable, presenting a more granular or porous texture, indicating the addition of the poly(3,4-ethylenedioxythiophene) (PEDOT) layer to the graphene layer used as a base. A more porous surface can provide a larger reaction surface area and more active sites for interaction with analytes. This can enhance the biosensor’s sensitivity and enable the detection of even very low concentrations of phenolic compounds. According to the specialized literature, a change in the morphological appearance of the surface is observed as the film thickness increases, transitioning from a porous surface to one with a globular aspect [24].

Following the immobilization of tyrosinase (Figure 1c), a significant change in the substrate morphology can be observed. This change is characterized by the formation of small clusters, as well as a surface texture that may suggest a configuration similar to cauliflower. This visual modification signals the potential interaction or chemical reaction between tyrosinase and the modified substrate, confirming the successful immobilization of the enzyme. In several cases, particle clustering can create favorable microenvironments for interaction with analytes, which can contribute to improving the biosensor’s sensitivity. It is important to continue additional investigations to fully understand the impact of this morphological change and to adapt experimental conditions and applications accordingly.

### 2.2. Electrochemical Characterization of the SPE/GPH/PEDOT/Ty

EIS (electrochemical impedance spectroscopy) is an efficient analysis technique that provides information about the charge transfer process at the interface between the electrode and electrolyte. The semicircle in the impedance spectrum reflects the kinetic-controlled electron transfer resistance (*R*_ct_), while the linear region that follows reveals the diffusion process at low frequencies [25]. The Nyquist plots of the SPE, SPE/GPH, SPE/GPH/PEDOT, and SPE/GPH/PEDOT/Tyr electrodes were analyzed individually, immersed in a solution containing 10^−3^ M [Fe(CN)_6_]^3−/4−^ with 10^−1^ M KCl as a redox probe, using EIS in a frequency range from 0.01 Hz to 1000 kHz.

In the impedance analysis, the size of the semicircle observed in the Nyquist plot provides information about the electron transfer resistance (*R*_ct_). It is evident that, compared to the unmodified SPE electrode, the electrodes modified with graphene (GPH) and GPH with PEDOT (SPE/GPH/PEDOT) exhibit better conductivity. This improvement is attributed to the significant enhancement in conductivity due to the presence of graphene and the polymer, resulting in a reduction in the value of *R*_ct_.

Additionally, it is observed that when tyrosinase (Tyr) is added to the surface of the modified electrodes, the charge transfer resistance for the SPE/GPH/PEDOT/Tyr electrodes is higher compared to the modified electrodes without Tyr. This observation suggests the successful immobilization of Tyr on the surface of the modified electrodes. The decrease in the electron transfer rate, caused by the presence of the enzyme, confirms the effectiveness of the immobilization process [26].

**Figure 2:**
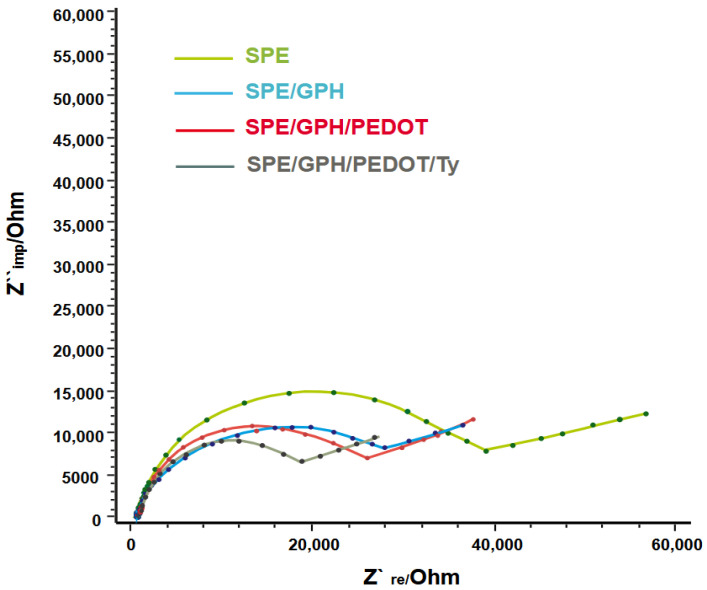
Electrochemical impedance spectroscopic measurements of SPE, SPE/GPH SPE/GPH/PEDOT, and SPE/GPH/PEDOT/Ty.

According to Figure 2, the *R*_ct_ values were obtained as 39,355 Ω, 27,242 Ω, 25,145 Ω, and 17,890 Ω for the SPE, SPE/GPH, SPE/GPH/PEDOT, and SPE/GPH/PEDOT/Ty electrodes, respectively. Both graphene and PEDOT increased the active surface area, contributing to an enhancement in conductivity and a decrease in *R*_ct_ values. The similarity of the Nyquist plots between the SPE/GPH and SPE/GPH/PEDOT electrodes can be explained by the fact that both electrodes were modified with graphene, a material that enhances conductivity and reduces *R*_ct_. Although PEDOT contributes to improving conductivity, the difference is not very significant, probably due to factors such as the interaction between the layers of graphene and PEDOT, the degree of uniform surface coverage with the polymer, or other aspects related to the structure, thickness, and composition of the deposited polymer film. Thus, the newly developed biosensor can exhibit superior performance compared to the SPE, SPE/GPH, and SPE/GPH/PEDOT electrodes for the detection of phenolic compounds.

The electrochemical behavior of the SPE/GPH, SPE/GPH/PEDOT, and SPE/GPH/PEDOT/Ty sensors was investigated using cyclic voltammetry in a solution containing 10^−3^ M potassium ferrocyanide and 10^−1^ M KCl. Cyclic voltammograms were recorded at a scan rate of 0.05 V/s in the potential range between −0.4 V and +1.0 V.

**Figure 3:**
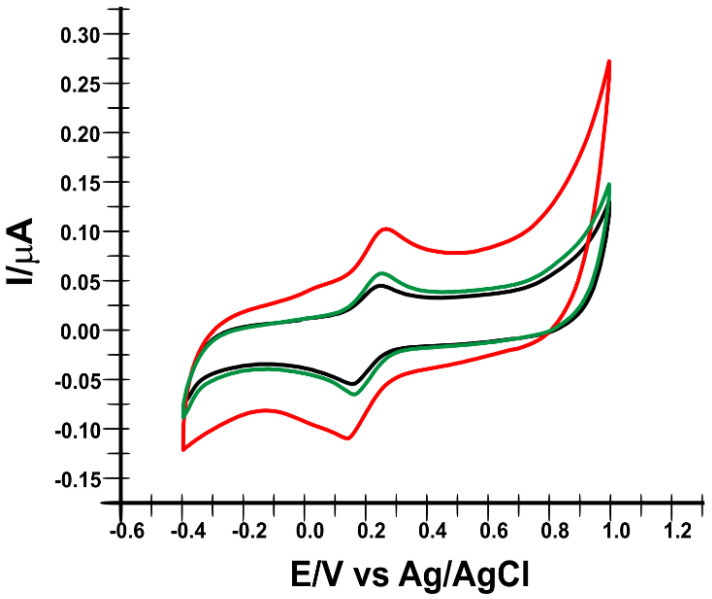
Cyclic voltammograms recorded by the SPE/GPH/PEDOT sensor in a solution of 10^−3^ M potassium ferrocyanide and 10^−1^ M KCl, constructed at different deposition times: 60 s (black line), 90 s (red line), and 120 s (green line).

In the experimental analysis of the modified SPE/GPH/PEDOT sensors, the influence of the deposition time of the PEDOT film on the electrochemical behavior of the modified sensor in the electroactive solution was examined. It was found that at a deposition time of 90 s, the peak intensity increased, and the cathodic peak potential shifted slightly to the left, compared to the response of sensors modified with deposition times of 60 s and 120 s. The higher peaks observed in the cyclic voltammogram at a deposition time of 90 s may indicate an improvement in the sensor’s ability to transfer electrons in the solution and detect electroactive species. This improvement could be attributed to the increased active surface of the PEDOT film and better accessibility of electroactive species. Although an increase in the thickness of the PEDOT film was recorded at a deposition time of 120 s, this could be associated with a higher density of defects or a more complex structural organization of the film. These aspects may negatively impact electron transfer and contribute to peaks with reduced intensities, as observed in Figure 3.

An excessive increase in thickness can lead to a longer response time of the sensor, limiting the diffusion of oxygen and electrolytes through PEDOT, and may affect its ability to rapidly detect changes in the analyte concentration. This aspect has been reported in other studies as well [27,28]. In other words, after 90 s, the increase in film thickness no longer brings significant improvements to the active surface but may lead to structural complications affecting the electrochemical behavior. It is crucial to find a balance between film thickness and its electrochemical properties to achieve the best response from the sensor. Therefore, for the construction of these sensors, the optimal deposition time for the PEDOT film is determined to be 90 s.

**Figure 4:**
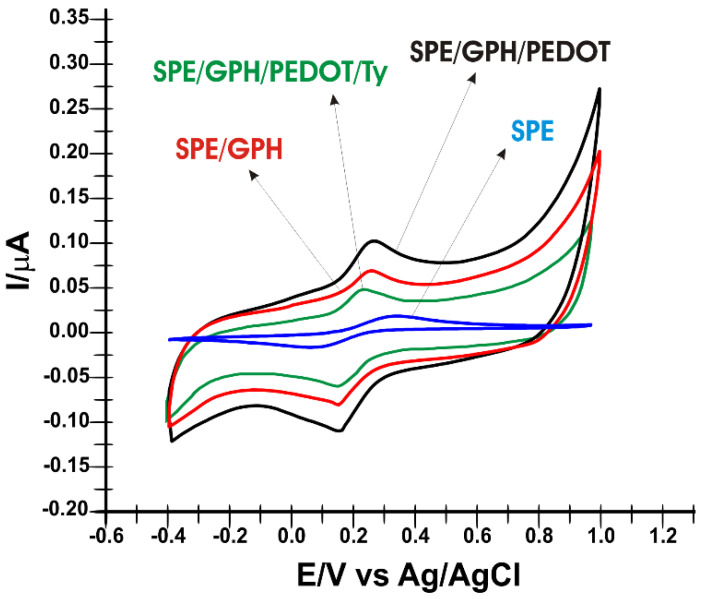
Cyclic voltammograms of SPE (blue line), SPE/GPH (red line), SPE/GPH/PEDOT (black line), and SPE/GPH/PEDOT/Ty (green line) in a solution of 10^−3^ M potassium ferrocyanide and 10^−1^ M KCl. Scan rate: 0.05 V/s.

In the next step, the electrochemical behavior of the four types of sensors—SPE, SPE/GPH, SPE/GPH/PEDOT, and SPE/GPH/PEDOT/Ty—was evaluated in a solution of 10^−3^ M potassium ferrocyanide and 10^−1^ M KCl, at a scan rate of 0.05 V/s. The obtained voltammograms for these sensors provide significant information about their electrochemical performance and the benefits brought by the modifications to the bare screen-printed electrode (Figure 4).

The SPE exhibits a standard behavior of the potassium ferrocyanide redox process. Modification with graphene leads to a significant increase in intensity and peak definition in the voltammogram. This indicates enhanced sensitivity of the sensor to the potassium ferrocyanide redox process, attributed to the electrocatalytic properties of graphene.

Depositing the PEDOT layer on the SPE/GPH sensor significantly improves the electrochemical performance of the sensor. This enhancement can be attributed to several factors. Firstly, PEDOT is known for its excellent conductivity, facilitating efficient electron transfer in electrochemical reactions. This results in increased current intensity, reflecting heightened sensor sensitivity. Secondly, the PEDOT film can increase the active surface area of the electrodes, providing more active sites for the electrochemical reaction to take place, explaining the significant increase in current intensity. Additionally, PEDOT provides functional groups that favor the redox process.

In the case of the tyrosinase biosensor, a decrease in peak intensity is observed compared to other modified sensors. This is because the tyrosinase enzyme does not necessarily offer better sensitivity in the potassium ferrocyanide redox process. However, reversibility is better compared to other modified sensors. Although the oxidation peak intensity is lower, the potential at which this oxidation peak occurs is also lower compared to modified sensors. This suggests a modification in the kinetics and mechanism of the potassium ferrocyanide oxidation reaction in the presence of the enzyme. The lower potential may indicate a faster electrochemical reaction or a better capacity of the biosensor to facilitate electron transfer between the enzyme and ferrocyanide. These aspects can significantly influence the performance and sensitivity of the biosensor in detecting phenolic compounds.

Table 1 presents the electrochemical parameters obtained from the cyclic voltammograms of the sensors immersed in a solution of 10^−3^ M potassium ferrocyanide—10^−1^ M KCl.

Summarizing the current stage of electrochemical characterization of the modified electrodes in a potassium ferrocyanide electroactive solution, the primary goal was to highlight the presence of tyrosinase, in the case of the biosensor. The next step will involve evaluating the behavior of these modified electrodes in solutions containing phenolic compounds. This step is crucial to determine the efficiency of the biosensor in the specific detection of these phenolic compounds, providing essential information for the further development and optimization of the device.

### 2.3. Electrochemical Behavior of Phenolic Compounds Using Modified Electrodes

In the first part of this section, the electrochemical behavior of the SPE/GPH/PEDOT/Ty biosensor was investigated in solutions containing catechol at a concentration of 10^−3^ M. This also involved optimizing key factors to properly assess the catechol redox process in this context.

**Figure 5:**
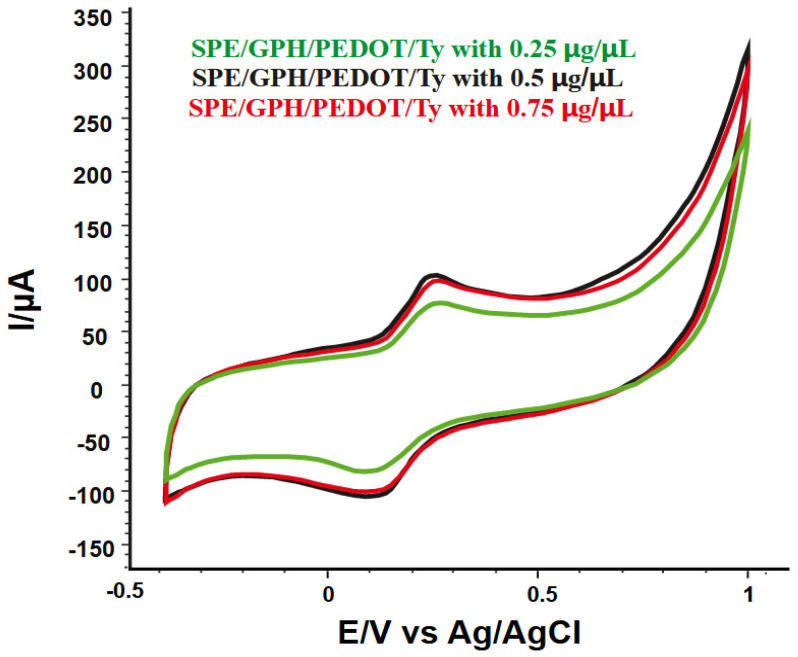
CVs of the biosensor with different concentrations of immobilized tyrosinase, immersed in a solution of 10^−3^ M catechol (pH 7.0).

Initially, the influence of the added enzymatic solution volume and, implicitly, the enzyme loading on the biosensor response were explored. This optimization was carried out by adding three distinct quantities. In the first approach, sequentially, dry and cross-linked (using 2% glutaraldehyde vapors), 5 µL of tyrosinase solution were added. The second biosensor was modified with 10 µL of enzyme solution, and the third with 15 µL of tyrosinase solution. The results revealed that an enzyme loading of 0.5 µg/µL (equivalent to 10 µL of enzyme solution) led to an optimized electrochemical response, characterized by higher peaks and better reversibility, suggesting a more efficient redox process (Figure 5), compared to the situation where only 0.25 µg/µL enzyme is immobilized. It is noteworthy that the biosensor with an enzyme loading of 0.75 µg/µL shows a response very similar to that with 0.5 µg/µL of tyrosinase, so an additional quantity that complicates and delays the manufacturing process is not justified. It may even lead to the saturation of active centers that can reduce the electrochemical response (as observed in Figure 5). Therefore, the decision was made to modify the sensor with a concentration of 0.5 µg/µL of tyrosinase.

The importance of rigorous pH control to ensure optimal working conditions for the SPE/GPH/PEDOT/Ty biosensor is a crucial aspect of this study. To achieve this goal, the redox process of catechol dissolved in various 10^−1^ M phosphate-buffered solutions was analyzed, covering the pH range between 6.5 and 7.5. This evaluation allows an assessment of how the pH of the environment influences the biosensor’s performance, ensuring its operation at maximum efficiency.

The obtained results highlighted that the current intensity related to catechol oxidation, through cyclic voltammetry using the SPE/GPH/PEDOT/Ty biosensor, did not undergo significant changes at pH values of 6.5, 7, and 7.5. This emphasizes the robustness of the developed biosensor to minor pH variations within this range, representing a significant advantage of the adopted construction method. However, pH 7.0 was chosen as the optimal value for further analyses, as the oxidation potential has a slightly lower value compared to other pH values in the analyte solution. This choice was also based on findings in the relevant literature and aligned with similar applications reported in this field, especially regarding the use of tyrosinase-based biosensors for the determination of phenolic compounds [19,29]. This behavior was similar for the other two phenolic compounds. Therefore, pH 7.0 was selected as the optimal working pH, emphasizing the utility and consistency of the biosensor in environmental applications.

In the next section of the study, the electrochemical behavior in solutions containing electroactive compounds, CAT, 3-methoxyCAT, and 4-methylCAT, was evaluated using cyclic voltammetry. For this analysis, only the modified sensors, SPE/GPH, SPE/GPH/PEDOT, and the SPE/GPH/PEDOT/Ty biosensor, which have already proven their efficiency and feasibility, were used. The experimental parameters involved a potential range between −0.4 and 1.0 V, with a scan rate of 0.05 V/s. This method allows the investigation of electrochemical reactions and the electroactive properties of these compounds, contributing to a deeper understanding of their behavior in the context of electrochemical applications.

**Figure 6:**
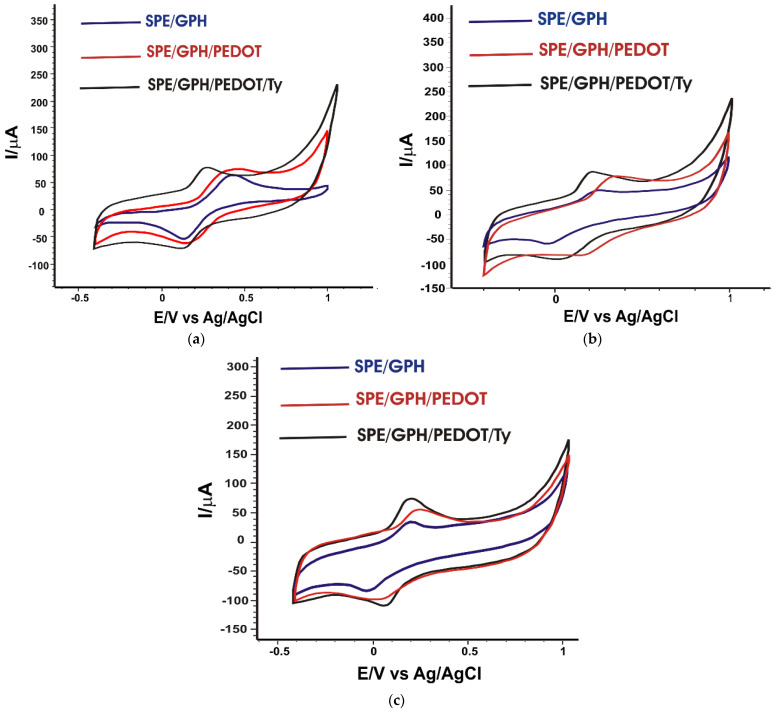
Cyclic voltammograms of the sensors in solutions of phenolic compounds at a concentration of 10^−3^ M, (**a**) CAT, (**b**) 3-methoxyCAT, and (**c**) 4-methylCAT.

Figure 6 shows the peaks corresponding to the redox process for the three phenolic compounds. It is evident that the biosensor records more intense and clear peaks compared to the other two modified sensors. This can be explained by the affinity of tyrosinase for the selected phenolic compounds, leading to much more sensitive and selective electrochemical responses. This is highlighted by both higher intensities and lower potentials, suggesting more efficient sensitivity and selectivity, likely due to the substrate’s affinity for the tested phenolic compounds.

**Scheme 1:**
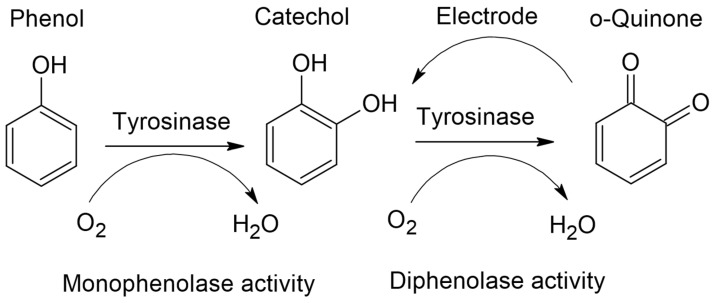
Catalytic mechanism of tyrosinase on phenolic compounds. Adapted from [31].

The redox process for the compounds CAT, 3-methoxyCAT, and 4-methylCAT involves a similar redox cycle. Tyrosinase catalyzes two types of reactions in the synthesis of melanin from catechol, a well-known substrate of the enzyme. The first reaction, called monophenolase activity, involves the hydroxylation of monophenols, generating o-diphenols [30]. The second activity, diphenolase activity, involves the oxidation of o-diphenols, forming o-quinone. Using molecular oxygen, both reactions generate o-quinone through the oxidation of diphenols (such as catechol) (Scheme 1) [31], thus being monitored voltamperometrically. Therefore, both catechol and its methylated and methoxylated derivatives undergo a similar redox process, involving two electrons in this redox reaction. The quinones resulting from these redox reactions are generally called benzoquinone, methoxybenzoquinone, and methylbenzoquinone.

The hydroxylation and oxidation process catalyzed by tyrosinase takes place in an enzyme’s active site, which is formed by a copper complex. This copper serves as a mediator for redox reactions, facilitating electron transfer between the chemical substances involved in the reaction and the enzyme. The tyrosinase-based biosensor operates by measuring the electrochemical changes resulting from the enzymatic reaction between phenolic compounds and tyrosinase. Tyrosinase catalyzes the conversion of phenolic compounds into redox intermediates, such as o-quinones, which can be detected with high sensitivity using electrochemical techniques, such as square wave voltammetry. When phenolic compounds, such as CAT, 3-methoxyCAT, and 4-methylCAT, come into contact with the active surface of the biosensor, they undergo the enzymatic reaction catalyzed by tyrosinase, resulting in the formation of o-quinones. This redox change is detected and measured, providing information about the concentration of phenolic compounds in the sample. Therefore, the tyrosinase-based biosensor offers a sensitive and specific method for detecting and quantifying phenolic compounds, including catechol, 3-methoxycatechol, and 4-methylcatechol, with promising applications in monitoring the quality of water and other environmental sources [30,31,32].

**Figure 7:**
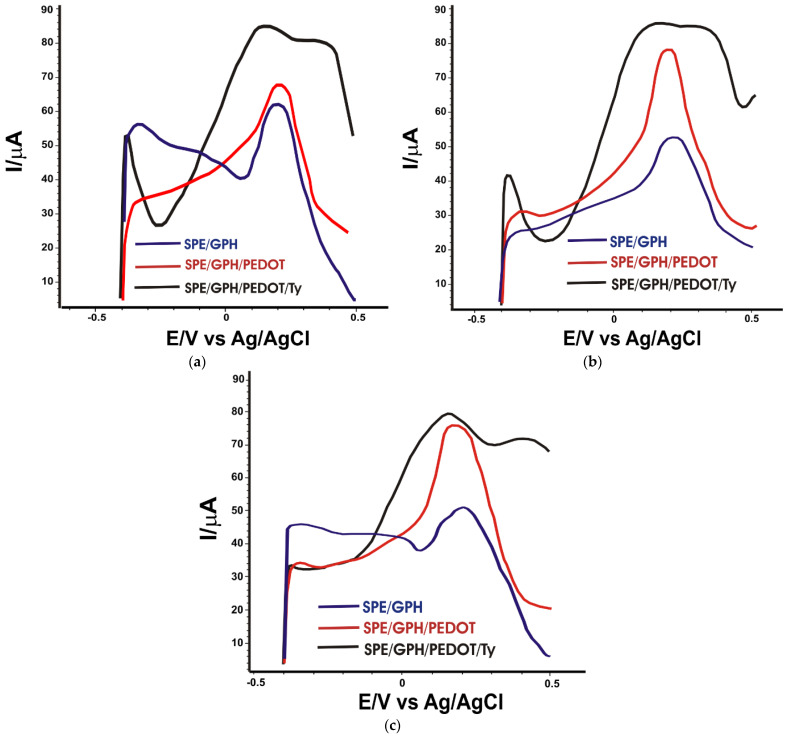
SWVs of the sensors in 10^−3^ M phenolic compound solutions, (**a**) CAT, (**b**) 3-methoxyCAT, and (**c**) 4-methylCAT.

Given the significant differences highlighted in cyclic voltammetry (CV) obtained with the biosensor, the decision was made to apply square wave voltammetry (SWV) for a more detailed and efficient analysis of redox processes. One of the notable advantages of square wave voltammetry (SWV) lies in its ability to provide increased sensitivity and resolution in detecting redox processes, due to its nature of minimizing background noise and emphasizing specific electrochemical signals (citation). After the prior optimization of parameters, including a frequency (f) set at 15 Hz, applied pulse (*E*_sw_) at 90 mV, and the applied potential range (*E*_i_–*E*_f_) between −0.4 and 0.5 V, square wave voltammetry (SWV) was implemented. Figure 7 presents the square wave voltammograms obtained with the three modified sensors in stock solutions of phenolic compounds, using a 10^−1^ M PBS electrolyte at pH 7. The observed difference in the SWV voltammogram, highlighting a more pronounced anodic peak associated with catechol oxidation in the biosensor’s case, can be explained by the improvements brought about by enzyme immobilization in terms of sensitivity and selectivity for redox processes.

Specifically, the way the biosensor was modified and optimized could have influenced the kinetics of the electrochemical reactions, leading to a more pronounced response associated with catechol oxidation. In the biosensor’s case, the appearance of an additional peak at around 0.4 V can be attributed to the enzyme-catalyzed oxidation of phenolic compounds in two distinct steps. This observation may be associated with the oxidation of an intermediate generated from the complex interaction between the phenolic compound and the tyrosinase enzyme. This mechanism has been previously described in the detection of p-ethylphenol using a tyrosinase-based biosensor [32].

A similar behavior was observed in the case of 3-methoxyCAT and 4-methylCAT compounds during electrochemical experiments. This similarity in electrochemical reactions can be attributed to the similar structural properties of these phenolic compounds, leading to comparable redox phenomena within the biosensor. Table 2 compares the values of anodic current intensities and oxidation potentials in the biosensor when using CV and SWV for the detection of the three phenolic compounds.

The differences in the oxidation peak intensity between CV and SWV can be attributed to the increased sensitivity and better resolution of the SWV technique, as well as the specific experimental parameter adjustments of this technique, which can influence the detection and recording of electrochemical signals. Also, the oxidation potential values are slightly lower when applying SWV, suggesting the increased sensitivity of the technique. In the case of both voltametric methods, the peaks are well-defined and reversible. Regarding the cathodic peaks, they have slightly lower current intensity values, so for calibration, the values of the anodic peaks will be recorded.

### 2.4. Calibration Curve and Detection Limit

The biosensor, having the best response in preliminary studies, was further tested for its ability to detect CAT, 3-methoxyCAT, and 4-methylCAT, phenolic compounds commonly encountered in water samples.

**Figure 8:**
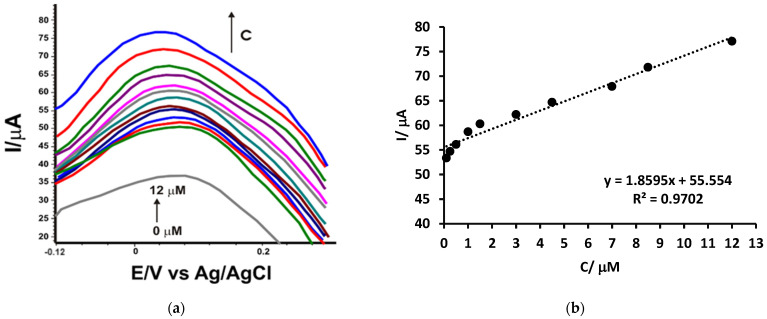

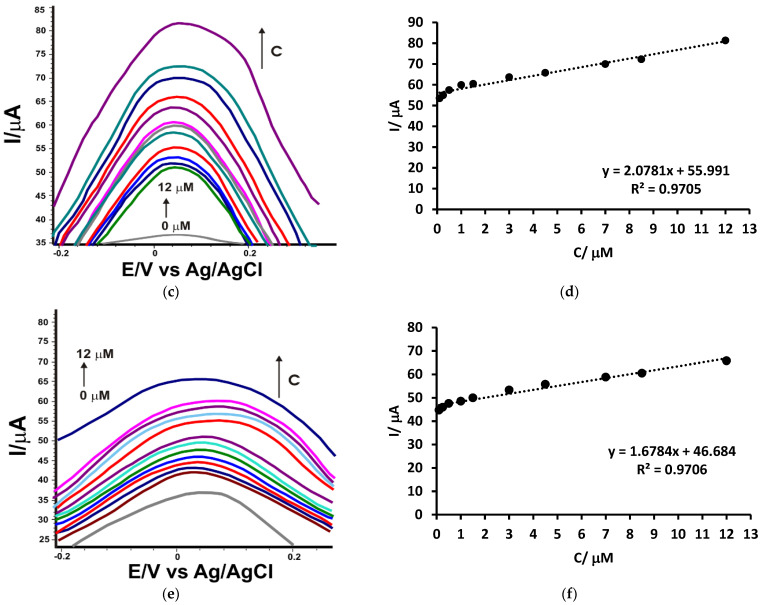
The zoom on the anodic peak of the voltammograms registered for solution of (**a**) CAT, (**c**) 3-methoxyCAT, and (**e**) 4-methylCAT in the concentration range 0–12 μM. Calibration curve in the range 0.1–12 μM for (**b**) CAT, (**d**) 3-methoxyCAT, and (**f**) 4-methylCAT.

For the construction of the calibration curve, square wave voltammograms of the biosensor were recorded in solutions of catechol, methoxy-catechol, and methyl-catechol in the concentration range of 0.01–12 µM. In Figure 8, an increase in the cathodic peak current can be observed with the increasing concentration of the phenolic compound, along with linear adjustments in the range of 0.1–12 µM.

Although linearity at very low concentrations was suboptimal compared to other studies [33], it is important to emphasize that we achieved good linearity over a much wider range of concentrations.

The observation of a slight shift in potential as the analyte concentration increases can be attributed to the presence of background current. This shift may be caused by the complex interactions between the electrodes and the test solution, which can be influenced by fluctuations in the surrounding environment that cannot be fully controlled. However, it is important to note that the difference between the potential values was not greater than 8%, indicating the stability and accuracy of the measurements overall.

Table 3 summarizes the characteristics of the newly developed biosensor for the selected phenolic compounds. The detection limit was defined as the lowest compound concentration that induces a reduction signal equivalent to 3 σ, where σ represents the standard deviation of the baseline current (noise) observed in the absence of the analyte. Similarly, the quantification limit was defined as the lowest concentration within the linear dynamic range [34].

For all phenolic compounds, SPE/GPH/PEDOT/Ty exhibited excellent performance in terms of linear range and sensitivity. The highest sensitivity was achieved using catechol as the substrate, while 3-methoxycatechol led to slightly reduced signals. These differences can be attributed to variations in the specificity of tyrosinase for the selected phenolic compounds.

To understand the enzymatic kinetics, a Lineweaver–Burk plot of 1/I (μA^−1^) vs. 1/[S] (μM^−1^) was constructed. From the Michaelis–Menten relationship, 1/I = 1/I_max_ + $K_{m}^{app}$/I_max_ [S] [35], $K_{m}^{app}$ and Imax were calculated for each substrate.

By graphically representing log [I/(I_max_ − I)] vs. log[S] [36], we obtained the Hill coefficient (h) from the slope of the line, indicated by its incline.

Table 4 presents the values of the kinetic parameters calculated for each phenolic compound, CAT, 3-methoxyCAT, and 4-methylCAT, using SPE/GPH/PEDOT/Ty.

The biosensor, SPE/GPH/PEDOT/Ty, exhibited a Hill coefficient for all three compounds close to 1, confirming Michaelis–Menten kinetics. The slightly higher value, especially for 3-methoxyCAT, indicates a positive cooperative effect between occupied active sites. In the case of CAT and 3-methylCAT, the interaction with the enzyme is not significantly influenced by the presence of other molecules.

On the other hand, the lower the Michaelis–Menten constant, the higher the affinity between Tyr and the substrate. If the biosensor has a low Michaelis–Menten constant, it can detect the analyte over a wide concentration range [37]. In the case of SPE/GPH/PEDOT/Ty, the $K_{m}^{app}$ values are suitable for all three phenolic compounds. A comparative study of tyrosinase-based biosensors reported in the literature is presented in Table 5. For the detection of catechol, SPE/GPH/PEDOT/Ty showed excellent analytical performance, with the lowest detection limit and the smallest value of $K_{m}^{app}$.

Given these favorable results, the SPE/GPH/PEDOT/Ty biosensor stands out as the suitable choice for quantitative determinations of catechol, 3-methoxycatechol, and 4-methylcatechol.

### 2.5. Study of Stability and Precision of the SPE/GPH/PEDOT/Ty

Stability and precision tests were conducted to assess the behavior of the SPE/GPH/PEDOT/Ty biosensor under different operating conditions. These tests aimed to verify the robustness and efficiency of the biosensor during repeated immersions in specific solutions, as well as during storage periods at controlled temperatures. By monitoring the intensity of the anodic current, the biosensor’s behavior over time was evaluated, providing essential information about its reliability and durability in practical applications. The results of these stability tests contributed to validating the performance of the SPE/GPH/PEDOT/Ty biosensor.

To examine the precision of the response to repeated recordings, the biosensor was repeatedly introduced (without renewing the surface) into solutions containing CAT, 3-methoxyCAT, and 4-methylCAT, all at a concentration of 5 × 10^−3^ M. This method involved immersing the same biosensor 10 times in the same solutions of phenolic compounds, and recordings were made through cyclic voltammetry with the same set parameters.

Additionally, the stability of the biosensor was analyzed over longer periods by immersing it in the same solutions for 10 consecutive days. The solutions of phenolic compounds were maintained at a constant concentration of 5 × 10^−3^ M, and the biosensor was stored at 4 °C between uses.

The test results highlighted similar performances of the biosensor during repeated immersions, with minor variations in the intensity of the anodic current (less than 6%). After a storage period of 30 days, the biosensor maintained significant stability in each solution (>75%). The optimal stability of the SPE/GPH/PEDOT/Ty biosensor exceeded that reported in the literature (less than 15 days) [44]. All recordings were performed in triplicate, and the results are presented in Table 6.

### 2.6. Reproducibility of the SPE/GPH/PEDOT/Ty Biosensor

Reproducibility was assessed through cyclic voltammetry recordings in a 5 × 10^−3^ M CAT solution, using two identically prepared biosensors. Both biosensors were employed under the same experimental conditions, demonstrating significant reproducibility. The difference between the intensities of the anodic currents did not exceed 8%.

### 2.7. Selectivity of the SPE/GPH/PEDOT/Ty Biosensor

**Figure 9:**
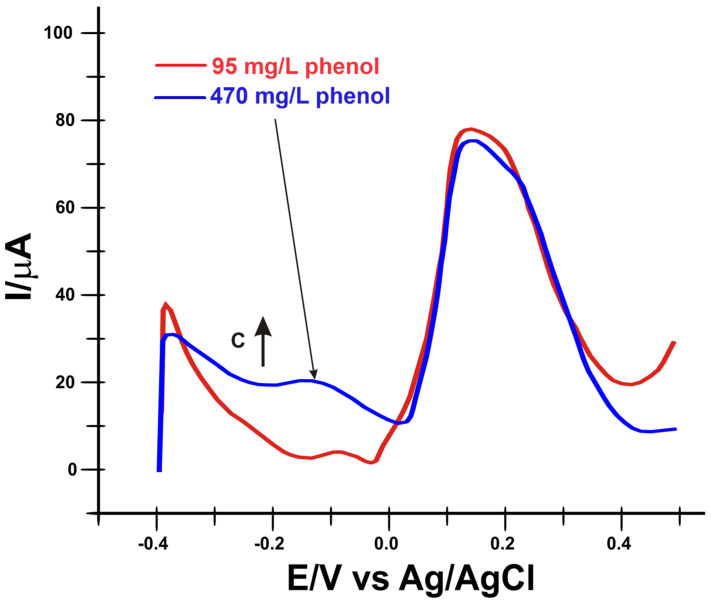
SWV of the SPE/GPH/PEDOT/Ty biosensor in solutions containing the same amount of CAT, 3-methoxyCAT, and 4-methylCAT (10^−3^ M) and different quantities of phenol (10^−3^ M and 5 × 10^−3^ M).

To assess the biosensor’s selectivity for the selected phenolic compounds in the study, the influence of phenol (10^−3^ M and 5 × 10^−3^ M), a compound present in water samples, was investigated. The concentrations of CAT, 3-methoxyCAT, and 4-methylCAT were 10^−3^ M, and the detection technique was SWV. The oxidation peak corresponding to the presence of phenol was at −0.082 V, a much lower potential compared to the oxidation potential of the diphenols present in the sample (Figure 9). Two different amounts of phenol were added, and the results confirmed that adding a concentration between 95 and 470 mg/L phenol did not modify the anodic current intensities of the three studied analytes by more than 8.2% (in the case of 3-methoxyCAT) at the higher phenol concentration. It is essential to mention that these added phenol concentrations in the samples significantly exceed the maximum allowable phenolic index limits in surface waters. Therefore, it is nearly impossible for such quantities of phenol to be present in real samples. However, there is a tendency for a higher amount of phenol to slightly inhibit the oxidation of the other phenolic compounds, especially in the case of 3-methoxycatechol. The results are presented in Table 7.

### 2.8. Real Sample Analysis

**Figure 10:**
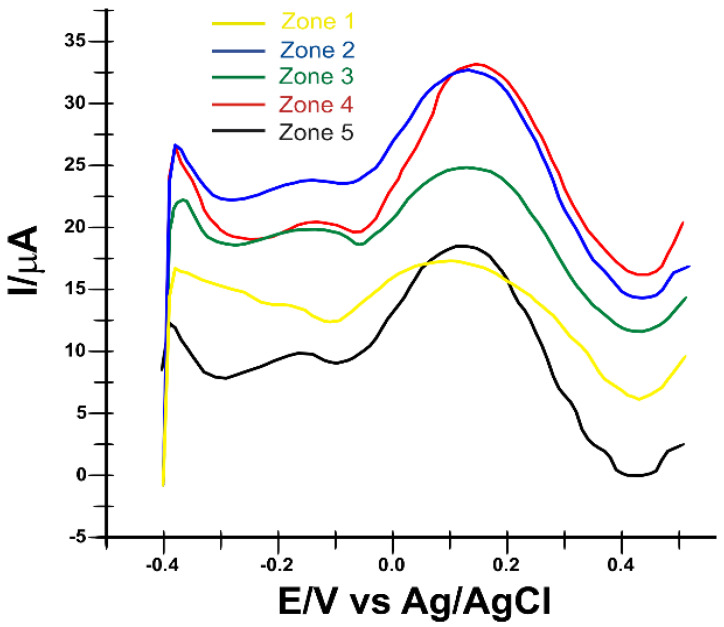
The square wave voltammograms of SPE/GPH/PEDOT/Ty immersed in water samples from the Danube River selected for analysis from different zones.

The detection of phenolic compounds in real samples, originating from five different regions along the Danube River, was conducted using square wave voltammetry. These samples were selected to investigate the potential natural contamination of the river waters. The potential range was from −0.4 to 0.5 V, the applied pulse was 90 mV, the scan rate was 7 mV/s, and the frequency was 15 Hz. The samples required prior preparation (see Section 2). Figure 10 presents the square wave voltammograms of SPE/GPH/PEDOT/Ty immersed in water samples from the Danube River, collected from five different zones selected for analysis.

With the aid of the anodic current intensities at the corresponding potential for CAT, 3-methoxylCAT, and 4-methylCAT, as well as the slope of the calibration curve, the quantities of the three compounds in the Danube River water samples were determined (Table 8).

The precision expressed as the relative standard deviation (RSD) was below 0.9%, indicating the efficiency of the method. The concentrations of CAT, 3-methoxyCAT, and 4-methylCAT in the Danube River waters from different zones do not exceed the maximum allowable limit for the phenolic index. Considering that these are the main phenolic compounds present in surface waters, we can assert that the water has satisfactory quality, and SPE/GPH/PEDOT/Ty is a useful and suitable tool for quantifying these analytes in environmental samples.

### 2.9. Validation of Results by the Standard Addition Method

Recovery studies were conducted by recording square wave voltammograms for the water sample from Zone 1, adding known concentrations of phenolic compounds using stock solutions of 10^−3^ M. By utilizing the anodic current intensities measured at the specific potential of each phenolic compound (E_CAT_—0.15 V, E_3-methoxyCAT_—0.14 V, and E_4-methylCAT_—0.17 V) and the calibration curve slope, their concentrations were calculated, as shown in Table 9.

The recovery values ranging between 98.07% and 101.45%, with a standard deviation coefficient (RSD) between 0.01% and 1.37%, indicate excellent performance and good precision of the SPE/GPH/PEDOT/Ty biosensor in the water analysis from Zone 1. These results suggest that the biosensor is reliable and precise in measuring the concentrations of these phenolic compounds in water, making it suitable for accurate water quality monitoring applications in this area.

## 3. Methods and Materials

### 3.1. Reagents and Solution

The chemicals used were of analytical purity, without the need for further purification steps. The 10^−1^ M phosphate-buffered solution at pH 7.0, used as the supporting electrolyte for electrochemical measurements, was prepared from Na_2_HPO_4_ and NaH_2_PO_4_ (Sigma-Aldrich, St. Louis, MO, USA), adjusting the pH with phosphoric acid.

3,4-Ethylenedioxythiophene, potassium ferrocyanide (FeCN), sodium sulfate (Sigma-Aldrich, St. Louis, MO, USA) dissolved in acetonitrile, and distilled water in a 1:1 ratio formed the solution necessary for electro-deposition.

Graphene powder (GPH) (Sigma-Aldrich, St. Louis, MO, USA) was dispersed in a mixture of dimethylformamide (DMF) (Sigma-Aldrich, St. Louis, MO, USA) and ultrapure water, forming the suspension needed for modifying the working electrodes.

Tyrosinase (EC:1.14.18.1), derived from mushrooms, in lyophilized form, with an activity of 5370 U mg^−1^, was acquired from Sigma Aldrich. For immobilization, a tyrosinase solution with a concentration of 50 μg/μL was prepared using PBS 10^−1^ M at pH 7.0 as the solvent.

Electrochemical impedance spectroscopy (EIS) and cyclic voltammetry (CV), applied for the electrochemical characterization of the modified sensors, required the preparation and use of a solution of potassium chloride (10^−1^ M) and potassium ferrocyanide (10^−3^ M) (Sigma-Aldrich, St. Louis, MO, USA) in a 1:1 ratio.

To obtain solutions of phenolic compounds (10^−3^ M), including phenol, CAT, 3-methoxyCAT, and 4-methylCAT, an appropriate amount of each compound was dissolved in a PBS 10^−1^ M solution at pH 7.0.

### 3.2. Apparatus

All solutions were prepared using ultrapure water obtained through the Millipore Milli-Q system (KGaA, Darmstadt, Germany), and for dissolving substances and homogenizing solutions, an Elmasonic S10H (Carl Roth GmbH, Karlsruhe, Germany) ultrasonic bath was employed. To adjust the pH to 7.0 for analyses with the tyrosinase-based biosensor and to 5.0 for the laccase-based biosensor, a pH meter from WTW in Weilheim, Germany, was used.

Electropolymerization and electrochemical measurements were conducted using a Biologic SP 150 potentiostat/galvanostat from Bio-Logic Science Instruments SAS, Seyssinet-Pariset, France. The acquisition, processing, and saving of data were performed using the EC–Lab Express software (version 5.52).

For sample analysis, a 50 mL electrochemical cell was utilized, incorporating reference electrodes and the counter electrode (Ag/AgCl reference electrode containing a 3 M KCl aqueous solution and a platinum plate of 1 cm^2^, respectively). The voltammograms obtained during the experiments were processed using CorelDraw10 software (version 10).

For studying surface morphology, a JEOL T-300 scanning electron microscope (Jeol, Akishima, Tokyo, Japan) was employed.

### 3.3. Modification of SPE Electrodes with GPH

The GPH suspension was prepared by dissolving 15 mg of graphene powder in 10 mL of solvent (dimethylformamide: water = 1:1). The suspension was subjected to ultrasound for 30 min at a frequency of 59 kHz. Subsequently, a well-defined volume (5, 10, or 15 μL) of this suspension was dispersed (using the drop and dry method) with the help of an Eppendorf micropipette onto the active surface (diameter 0.4 cm) of the commercially available carbon screen-printed electrode (SPE) (geometric area of 0.125 cm^2^). The solvent completely evaporated after approximately 60 min in a desiccator. Until the electro-deposition of the PEDOT film, the graphene-modified sensors (SPE/GPH) were stored at 8 °C but not exceeding 48 h.

### 3.4. Synthesis of the PEDOT Film

**Figure 11:**
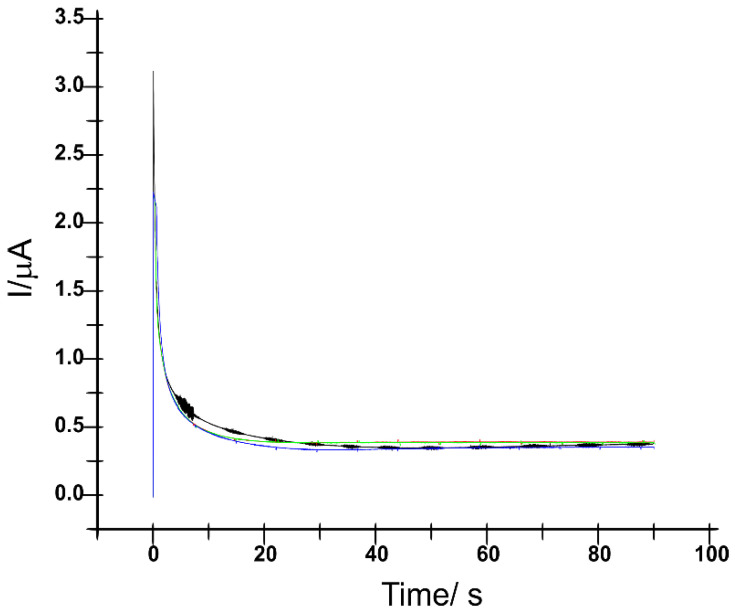
Current versus time curves registered during the electrosynthesis of four different sensors prepared in the same conditions.

The PEDOT film was deposited through chronoamperometry at a constant potential of 1 V using the same three-electrode electrochemical cell described earlier. The deposition of the PEDOT film was carried out in a single polymerization step by immersing the SPE/GPH in a solution containing acetonitrile as the solvent, the monomer (EDOT), and the doping agent (FeCN 0.05 M), at a potential of 1 V, varying the deposition time (60, 90, and 120 s) for optimization. At the end of this step, the SPE/GPH/PEDOT sensors were obtained. The chronoamperograms obtained from the electropolymerization process were presented as current intensity versus time. The chronoamperograms of four identically modified sensors (deposition time 90 s) were compared to determine the reproducibility of electropolymerization (Figure 11). Minimal differences were observed, with the calculated coefficient of variation being 3.35% between the chronoamperograms of sensors prepared under the same conditions.

To highlight the influence of the deposition time on the thickness of the PEDOT layer deposited on the surface of the screen-printed electrode modified with GPH, the ratio was calculated using Equation (1) [46,47]:

(1) $$d=\frac{Q\;\times \;M}{n\;\times \;F\;\times \;A\;\times \;\rho }$$

where

Q is the charge (C) obtained during the oxidation process

n is the number of electrons involved in the oxidation reaction

F is the Faraday constant (96,485.33 C/mol)

A is the active surface area of the sensor (cm^2^)

ρ is the density of PEDOT (1.011 g/cm^3^).

This calculation allows for determining the thickness of the PEDOT layer based on the electrochemical parameters.

The value of d was calculated for three modified sensors (SPE/GPH/PEDOT) at different deposition times (60 s, 90 s, and 120 s). The results are presented in Table 10.

It can be observed that there is a considerable difference in the increase of PEDOT film thickness when the deposition time increases from 60 s to 90 s compared to the increase from 90 s to 120 s, where the film thickness only increases by 0.18 mm. This suggests that an increase in deposition time beyond 90 s may not be justified, as the thickness of the PEDOT film does not change significantly, likely due to saturation of the active surface of the SPE/GPH sensor. However, the influence of the deposition time will also be studied through the sensor response in an electroactive solution.

### 3.5. Preparation of Enzyme Electrodes

To construct the biosensor, electrodes previously modified by the electro-deposition of a conductive polymer, SPE/GPH/PEDOT, were used. Various volumes (5, 10, and 15 μL) of the tyrosinase solution (50 μg/μL) were added to the active surface of the electrodes to test the influence of the enzyme quantity on the electrochemical response of the biosensor. After drying, all biosensors (SPE/GPH/PEDOT/Ty) were exposed to 2% glutaraldehyde vapors (in 10^−1^ M PBS at pH 7) for 20 min at room temperature for cross-linking [36]. After this step, the biosensors were dried at a temperature of 10 °C and then, rinsed with PBS to remove any unbound enzyme from the surface of the PEDOT film. Subsequently, they were dried again at 10 °C and stored at 4 °C until the time of use, but not exceeding 72 h [48].

### 3.6. Samples Tested

The tested samples were collected from the Danube River from different zones (Zone 1, Zone 2, Zone 3, Zone 4, Zone 5) within the city of Galati, on the same day. The samples required minimal pre-treatment, undergoing filtration through blue band filter paper to remove impurities. Each filtrate was mixed with an equal amount of ultrapure water and then subjected to electrochemical testing. The final sample volume was 50 mL.

## 4. Conclusions

This study proposed and validated the effectiveness of an innovative biosensor, SPE/GPH/PEDOT/Ty, for the detection and quantification of phenolic compounds such as catechol, 3-methoxycatechol, and 4-methylcatechol in water samples from the Danube River. The biosensor demonstrated exceptionally low detection limits (LODs) for all phenolic compounds, with values recorded at 8.63 × 10^−10^ M for catechol, 7.72 × 10^−10^ M for 3-methoxycatechol, and 9.56 × 10^−10^ M for 4-methylcatechol. These values underscore the biosensor’s ability to accurately detect even subtle variations in trace compound parameters.

Furthermore, the biosensor’s enhanced sensitivity facilitated effective detection and quantification in river water samples. This heightened sensitivity is crucial for ensuring the reliable monitoring of water quality, particularly in environments where phenolic compounds may exist in minute concentrations. Overall, these electroanalytical results further affirm the biosensor’s efficacy and its potential for practical applications in water quality monitoring.

Recovery studies confirmed the exceptional precision of the biosensor, with recoveries ranging from 98.07% to 101.45% and coefficients of variation between 0.01% and 1.37%. Its selectivity was highlighted in the presence of phenol, and tests demonstrated its stability during repeated immersions and long-term storage.

The analysis of the Danube River water in different zones emphasized the applicability of the biosensor in real environmental conditions, with measured concentrations below the maximum allowable limits, indicating satisfactory water quality. The use of the standard addition method for validation strengthened the robustness of the biosensor and confirmed the consistency of the obtained data (recovery exceeding 98.07%). The concentrations found in real samples of phenolic compounds fall within the permissible limits according to the standards, reflecting satisfactory water quality.

The reproducibility of the biosensor was confirmed (the differences between the responses of two identically prepared biosensors did not exceed 8% through repeated recordings), highlighting its potential in water quality monitoring. In conclusion, the SPE/GPH/PEDOT/Ty biosensor appears as a promising and efficient tool for the detection of phenolic compounds in surface waters, providing precise, stable, and relevant results for practical water quality monitoring applications.

## Figures and Tables

**Table 1:** Electrochemical parameters obtained for the four sensors.

Sensor	ΔE (V)	E_1/2_ (V)	I_a_ (μA)	I_c_ (μA)	E_a_ (V)	E_c_ (V)	I_c_/I_a_
SPE	0.229	0.215	24.30	−23.5	0.330	0.101	0.96
SPE/GPH	0.104	0.200	74.25	−73.45	0.252	0.148	0.97
SPE/GPH/PEDOT	0.110	0.206	102.15	−110.5	0.262	0.151	1.08
SPE/GPH/PEDOT/Ty	0.077	0.191	51.120	−38.62	0.230	0.153	0.75

ΔE—Potential difference, E_1/2_—Half-wave potential, I_a_—Anodic peak current, I_c_—Cathodic peak current.

**Table 2:** Parameters related to the oxidation of phenolic compounds obtained with SPE/GPH/PEDOT/Ty when applying the two voltametric techniques.

**CV**			
	CAT	3-methoxyCAT	4-methylCAT
**I_a_ (μA)**	77.45	84.65	71.15
**E_a_ (V)**	0.25	0.1	0.21
**SWV**			
**I_a_ (μA)**	85.15	86.25	79.15
**E_a_ (V)**	0.15	0.14	0.17

**Table 3:** The characteristics of the developed biosensor regarding phenolic substrates (CAT, 3-methoxyCAT, 4-methylCAT).

Analyte	CAT	3-methoxyCAT	4-methylCAT
SPE/GPH/PEDOT/Ty *E*_i_–*E*_f_: −0.4–0.5 V vs. Ag/AgCl, *E*_sw_ = 0.09 V, *P*_w_ = 50 ms			
Sensitivity (A mol^−1^ L)	1.859 × 10^−6^	2.078 × 10^−6^	1.678 × 10^−6^
Limit of detection (mol L^−1^)	8.63 × 10^−10^	7.72 × 10^−10^	9.56 × 10^−10^
Limit of quantification (mol L^−1^)	2.88 × 10^−9^	2.57 × 10^−9^	3.18 × 10^−9^
Linear range (mol L^−1^)	1 × 10^−7^–1.2 × 10^−5^		
R^2^	0.9702	0.9705	0.9706

**Table 4:** The main kinetic parameters calculated for each phenolic compound using SPE/GPH/PEDOT/Ty.

Parameter/Analyte	I_max_ (μA)	$K_{m}^{app}$ (μM)	Hill Coefficient (h)
CAT	61.72	0.327	1.02
3-methoxyCAT	62.5	0.368	1.16
4-methylCAT	52.08	0.817	1.11

**Table 5:** Contrast of the analytical parameters acquired for the proposed amperometric method with those from other electroanalytical approaches documented in the literature for catechol determination.

Electrode	Linear Range (M)	LOD (M)	Sensitivity (A × mol^−1^ L)	$K_{m}^{app}$ (M)	Ref
Tyr-AuNPs-DHP/GCE	2.5–95 × 10^−6^	1.7 × 10^−7^	0.115	0.0002	[38]
Tyr-PO4–PPy/Pt	10–120 × 10^−6^	8.4 × 10^−7^	0.047	0.0000802	[36]
Tyr-GA/CBPE	5.0 × 10^−9^–4.8 × 10^−8^ 4.8 × 10^−8^–8.5 × 10^−6^	1.5 × 10^−9^	-	1.2 × 10^−5^	[39]
SPE/IrOx/PEDOT/Tyr	0.05 × 10^−6^−10.65 × 10^−6^	1.7 × 10^−8^	3.017 × 10^−6^	5.2 × 10^−5^	[31]
Tyr/GO/GA/GCE	5 × 10^−8^−5 × 10^−5^	3 × 10^−8^	0.34 × 10^−6^	16.99 × 10^−5^	[15]
Tyr-ND-PS/GCE	5–740 × 10^−6^	0.39 × 10^−6^	-	-	[40]
TMCG	1−30 × 10^−6^	2.60 × 10^−7^	0.057	22.5 × 10^−6^	[41]
Tyr-CNTs-Glu/NDs-SS/GCE	0.03–30 × 10^−6^	9.8 × 10^−9^	-	205.17 × 10^−6^	[20]
WS_2_-COOH nanotube + GCE	0.6–70 × 10^−6^	-	0.0107	183 × 10^−6^	[42]
Tyr-Fe_3_O_4_/PPy@TiO_2_@*n*-C_20_ MEPCM-modified GCE	1–10 × 10^−3^	3.4 × 10^−6^	0.102 × 10^−6^	-	[43]
SPE/GPH/PEDOT/Ty	1 × 10^−7^–1.2 × 10^−5^	8.63 × 10^−10^	1.859 × 10^−6^	0.254 × 10^−6^	This work

PPy: polypyrrole, DHP: Dihexadecylphosphate, GA/GLU: glutaraldehyde; GO: graphene oxide, GCE: glassy carbon electrode, NDs: Carbon nanodiamonds (NDs); PS: potato starch, IrOx: iridium (IV) oxide, TMCG: tyrosinase immobilized chitosan magnetite nanobiocomposite film, NDs: nanodiamonds soluble starch, TiO_2_@*n*-C20 MEPCM: microencapsulated *n*-eicosane with a TiO_2_ shell.

**Table 6:** Stability and precision of the biosensor in 5 × 10^−3^ M phenolic compound solutions.

Solution 5 × 10^−3^ M	RSD % 10 Consecutive Scan	RSD % 10 Consecutive Days (One Scan per Day)
CAT	1.63	1.82
3-methoxyCAT	1.56	2.08
4-methylCAT	2.61	2.87

**Table 7:** Results obtained for the determination of CAT, 3-methoxyCAT, and 4-methylCAT in the presence of phenol.

Phenolic Compound Present in Sample	Ratio	Recovery (%)	RSD (%)	Ratio	Recovery (%)	RSD (%)
**Interfering compound** Phenol						
CAT	1:1	94.06	4.32	1:5	93.00	5.12
3-methoxyCAT		93.04	5.09		91.88	5.98
4-methylCAT		99.17	0.58		95.07	3.57

**Table 8:** Concentrations of phenolic compound (*n* = 3) in Danube water obtained by the voltammetric method.

Sample	c (μM)	c (cg/L)	RSD (±%)	Phenolic Index (Order No. 161 Dated 16 February 2006) (cg/L) [45]
CAT				5
Zone 1	9.047	0.099	0.877	
Zone 2	17.412	0.195	0.163	
Zone 3	13.076	0.147	0.320	
Zone 4	17.783	0.200	0.271	
Zone 5	9.774	0.109	0.270	
3-methoxyCAT				
Zone 1	8.147	0.114	0.271	
Zone 2	15.644	0.324	0.116	
Zone 3	11.833	0.245	0.426	
Zone 4	15.842	0.328	0.335	
Zone 5	8.806	0.182	0.362	
4-methylCAT				
Zone 1	9.287	0.126	0.545	
Zone 2	19.123	0.264	0.406	
Zone 3	14.326	0.197	0.542	
Zone 4	19.290	0.122	0.277	
Zone 5	10.423	0.066	0.230	

**Table 9:** Quantitative results obtained for the detection of phenolic compound by the standard addition method.

Sample	CAT (μM) Initial	CAT (μM) Added	CAT (μM) Found	Recovery (%)
Zone 1	9.047	2	10.97	99.30 ± 0.49
		4	13.09	100.32 ± 0.23
		7	16.28	101.45 ± 1.01
	3-methoxyCAT (μM) Initial	3-methoxyCAT (μM) Added	3-methoxyCAT (μM) Found	Recovery (%)
	8.147	2	10.00	98.55 ± 1.03
		4	11.98	98.62 ± 0.97
		7	15.32	101.14 ± 0.80
	4-methylCAT (μM) Initial	4-methylCAT (μM) Added	4-methylCAT (μM) Found	Recovery (%)
	9.287	2	11.07	98.07 ± 1.37
		4	13.29	100.02 ± 0.01
		7	16.08	98.72 ± 0.90

**Table 10:** Variation in electric charge and PEDOT film thickness depending on the applied deposition time for the SPE/GPH/PEDOT sensor.

t (s)	Q (C)	d (μm)
60	24.57	2.86 ± 0.03
90	38.54	4.50 ± 0.04
120	40.21	4.68 ± 0.04

## Data Availability

Data is contained within the article.
